# Distribution of the Noradrenaline Innervation and Adrenoceptors in the Macaque Monkey Thalamus

**DOI:** 10.1093/cercor/bhab073

**Published:** 2021-05-18

**Authors:** Isabel Pérez-Santos, Nicola Palomero-Gallagher, Karl Zilles, Carmen Cavada

**Affiliations:** Departamento de Anatomía, Histología y Neurociencia, Facultad de Medicina, Universidad Autónoma de Madrid (UAM), Calle Arzobispo Morcillo 4, 28029 Madrid, Spain; Institute of Neuroscience and Medicine (INM-1), Research Centre Jülich, 52425 Jülich, Germany; Department of Psychiatry, Psychotherapy and Psychosomatics, Medical Faculty, RWTH Aachen University, 52074 Aachen, Germany; C. & O. Vogt Institute for Brain Research, Heinrich-Heine-University, 40225 Düsseldorf, Germany; Institute of Neuroscience and Medicine (INM-1), Research Centre Jülich, 52425 Jülich, Germany; C. & O. Vogt Institute for Brain Research, Heinrich-Heine-University, 40225 Düsseldorf, Germany; JARA-BRAIN, Jülich-Aachen Research Alliance, 52425 Jülich, Germany; Departamento de Anatomía, Histología y Neurociencia, Facultad de Medicina, Universidad Autónoma de Madrid (UAM), Calle Arzobispo Morcillo 4, 28029 Madrid, Spain

**Keywords:** dopamine-beta-hydroxylase, nonhuman primates, noradrenergic receptors, norepinephrine, norepinephrine transporter

## Abstract

Noradrenaline (NA) in the thalamus has important roles in physiological, pharmacological, and pathological neuromodulation. In this work, a complete characterization of NA axons and Alpha adrenoceptors distributions is provided.

NA axons, revealed by immunohistochemistry against the synthesizing enzyme and the NA transporter, are present in all thalamic nuclei. The most densely innervated ones are the midline nuclei, intralaminar nuclei (paracentral and parafascicular), and the medial sector of the mediodorsal nucleus (MDm). The ventral motor nuclei and most somatosensory relay nuclei receive a moderate NA innervation. The pulvinar complex receives a heterogeneous innervation. The lateral geniculate nucleus (GL) has the lowest NA innervation.

Alpha adrenoceptors were analyzed by in vitro quantitative autoradiography. Alpha-1 receptor densities are higher than Alpha-2 densities. Overall, axonal densities and Alpha adrenoceptor densities coincide; although some mismatches were identified. The nuclei with the highest Alpha-1 values are MDm, the parvocellular part of the ventral posterior medial nucleus, medial pulvinar, and midline nuclei. The nucleus with the lowest Alpha-1 receptor density is GL. Alpha-2 receptor densities are highest in the lateral dorsal, centromedian, medial and inferior pulvinar, and midline nuclei.

These results suggest a role for NA in modulating thalamic involvement in consciousness, limbic, cognitive, and executive functions.

## Introduction

Noradrenaline (NA) is an important modulatory neurotransmitter (Poe et al. 2020), often associated with signal-to-noise amplification in sensory systems (Foote et al. 1975; Ego-Stengel et al. 2002; Kuo and Trussell 2011) and to cognition modulation (Sara 2009). The role of NA in the thalamus is particularly relevant because most sensory inputs reaching the cortex are transmitted by and regulated within the first order sensory thalamic nuclei; and because higher brain functions, including learning and memory, hinge on the higher order thalamic nuclei involved in extensive cortico-thalamo-cortical pathways (Sherman 2016).

The role of thalamic NA has been studied, in rodents, in the context of somatosensory processing and signal-to-noise amplification (Hirata et al. 2006; Moxon et al. 2007; Wimmer et al. 2015), motor control (Grasso et al. 2006), and sensorimotor gating related to executive disorders (Alsene et al. 2011).

Thalamic NA is additionally relevant because many drugs act through the brain noradrenergic system: atomoxetine, a selective NA reuptake inhibitor used to treat attention deficit hyperactivity disorder (ADHD) (Hammerness et al. 2009); clonidine, an Alpha-2 adrenoceptor agonist used in the treatment of many conditions such as opiate withdrawal, posttraumatic stress disorder (PTSD) (Boehnlein and Kinzie 2007), and ADHD and Tourette’s syndrome (Tourette’s Syndrome Study 2002; Roessner et al. 2011); prazosin, an Alpha-1 adrenoceptor antagonist used in PTSD (Boehnlein and Kinzie 2007; Simon and Rousseau 2017); or dexmedetomidine, a highly selective Alpha-2 adrenoceptor agonist widely used as anesthetic, as it provides both sedation and analgesia without respiratory depression (Panzer et al. 2011; Song et al. 2017). In particular, thalamic functional connectivity alterations have been described under the influence of dexmedetomidine, and these alterations correlate with the unconsciousness it induces (Akeju et al. 2014). It is important to understand the brain sites and mechanisms through which the pharmacological noradrenergic compounds exert their effects.

NA deficits have been reported in the thalamus of Parkinson’s disease (PD) patients (Pifl et al. 2012; Nahimi et al. 2018) and in a primate model of PD (Pifl et al. 2013). Remarkably, the NA loss in the thalamus appears even higher than the dopamine loss. There are also reports on alterations in NA receptors in the brain of parkinsonian patients, including the prefrontal cortex (Cash et al. 1984). Besides, atomoxetine, the NA reuptake inhibitor mentioned above, improves executive dysfunction and response inhibition in PD patients (Marsh et al. 2009; Rae et al. 2016).

Despite the interest of the NA system in the primate thalamus, both in brain function, dysfunction, and pharmacological intervention, only descriptions with low-resolution techniques or fragmented analyses have been reported to date. In 1982, a description of the catecholaminergic fibers was made, using the Falck-Hillarp method: a high concentration of catecholaminergic fibers in the paramedian thalamic region of the monkey was reported (Tanaka et al. 1982). Later, anatomical studies based on retrograde tracers and immunohistochemistry against the NA synthesizing enzyme dopamine-beta-hydroxylase (DβH) were published in the context of different functional systems, such as the visual system (Morrison and Foote 1986), the somesthetic-nociceptive system (Westlund et al. 1990; Vogt et al. 2008), or together with other diencephalic structures (Ginsberg et al. 1993). These studies suggest heterogeneity in the distribution of the NA axons throughout the thalamic nuclei. The dopaminergic innervation is also quite heterogeneous among the various thalamic nuclei, both in monkeys and in humans (Sanchez-Gonzalez et al. 2005; Garcia-Cabezas et al. 2007).

The distribution of NA in the human thalamus has been described using high performance liquid chromatography with electrochemical detection (Oke et al. 1997). This study described a moderate NA concentration in all thalamic nuclei, with a higher concentration in anterior, medial and ventral regions; a lower concentration in posterior nuclei, and the lowest concentration in the lateral geniculate nucleus (GLd) (Oke et al. 1997). Because of the employed technique, no detailed study of each thalamic nucleus was possible. More recently, using autoradiography methods to detect the noradrenaline transporter (NET), the highest binding levels were found in the most medial regions of the macaque thalamus, as well as in some intralaminar nuclei (Smith et al. 2006). In humans, positron emission tomography (PET) studies to map the NET did not provide enough spatial resolution to distinguish its distribution within the various thalamic nuclei (Takano et al. 2008).

Descriptions of adrenoceptor location have been made in the nonhuman primate brain and also in the human brain. Some of them included the thalamus within the studied areas; however, the thalamus was considered as a whole, with no attention to differences between the thalamic nuclei (Reznikoff et al. 1986; Palacios et al. 1987; Nahimi et al. 2015).

In this study, we provide a comprehensive description of the macaque monkey thalamic NA system, including NA axons and Alpha adrenoceptor distributions. Alpha adrenoceptors are the most relevant adrenoceptors to the physiology, pathology, and pharmacology of the central nervous system (Samuels and Szabadi 2008a, 2008b). We provide detailed maps of the distributions of NA axons and receptors in the coronal plane, which will serve as a tool of reference for further electrophysiological, pharmacological, and neuroimaging studies.

## Materials and Methods

### NA-Axon Maps

Tissue from 2 monkeys, 5.5 ± 0.5 years of age, was used to generate the NA axons maps: a female *Macaca nemestrina,* and a male *Macaca mulatta*. Sections of similar thalamic coronal levels from the 2 monkeys were inmmunostained for 2 markers of the NA phenotype, DβH and NET, in serial sections.

The monkeys were housed in an animal room under standard conditions and treated in accordance with European guidelines (86/609/EEC and 2003/65/EC European Council Directives). The experiments were approved by the Committee for Research Ethics of the Universidad Autónoma de Madrid.

#### Tissue Preparation

The animals were deeply anesthetized with sodium pentobarbital and perfused through the ascending aorta with saline, followed by 4% paraformaldehyde in phosphate buffer (PB, 0.1 M, pH 7.4) and a series of PB sucrose solutions (5–20%). The brains were stereotaxically blocked in the coronal plane in 1-cm-thick blocks, taking the interaural plane as coronal plane 0. Blocks were cryoprotected in a 30% PB sucrose solution at 4°C until they sank and were cut in 40 μm coronal sections on a freezing microtome. A total of 10 series of sections were collected, so that the distance between sections within 1 series was 400 μm. Series 2, 5, and 8 were processed for acetylcholinesterase (AChE) histochemistry (Cavada et al. 1995), cresyl-violet staining, and myelin silver staining (Gallyas 1979), respectively. One of the in-between series was processed for the immunohistochemistry of interest (DβH or NET), so that the immunostained sections were adjacent or very close to sections processed to identify the thalamic nuclei. The sections to be immunostained were stored at −20°C in an ethylene–glycol solution until immunohistochemical processing.

#### DβH and NET Immunostaining

After washing the ethylene-glycol, the 40 μm formaldehyde-fixed coronal sections containing the thalamus were immunostained using antibodies against DβH and NET, which specifically reveal the NA phenotype. DβH might also immunostain adrenergic fibers, but they are scarce in the macaque monkey thalamus (Rico and Cavada 1998). NET is expressed only in NA cells (Lorang et al. 1994; Schroeter et al. 2000). None of the 2 markers would reveal the dopamine phenotype. Immunostaining was conducted as follows: 1) Endogenous peroxidase inhibition: 20 min at room temperature in a solution of 3% H_2_O_2_ and 10% methanol in tris buffer saline (TBS); 2) Antigen retrieval: 60 min at 90°C in sodium citrate buffer (pH 6.00) and 60 min at room temperature to cool down; 3) Preincubation: 3 h at room temperature in a blocking solution of TBS containing 20% serum from the species where the secondary antiserum was raised (donkey serum for DβH staining, and rabbit serum for NET staining), 5% bovine serum albumin (BSA), and 0.4% Triton X-100; 4) Incubation with the primary antibody: sheep polyclonal anti-DβH, AB1537, 1:1000 (Chemicon International, USA); or mouse monoclonal anti-NET, MAB5620, 1:1000 (EMD Millipore Corporation, USA) diluted in TBS containing 20% serum, 5% BSA, and 0.3% Triton X-100, for 60 h at 4°C; 5) Incubation with the secondary antiserum: biotinylated donkey antisheep, AP184B, 1:400; (Chemicon International, USA); or biotinylated rabbit antimouse, ab97044, 1:1000, (Abcam, Cambridge, UK) diluted in TBS containing 20% serum, 5% BSA, and 0.3% Triton X- 100, for 2 h at room temperature; 6) Incubation in Avidin/Biotin complex Vectastain Elite (Vector Labs, USA) diluted in TBS, for 45 min at room temperature; and 7) Development following the glucose oxidase-DAB-nickel method (Shu et al. 1988).

Rinses in tris buffer or TBS were made between steps, except between preincubation and incubation with the primary antibody.

Negative and positive controls were run parallel to all of the experimental immunohistochemistry series. The positive controls were cortical areas where noradrenergic axons should be present. Negative controls consisted of substituting the primary antibodies with sera from the species in which they had been raised. Also, a series from the brainstem and a series covering the whole brain were performed to check that only the putative noradrenergic neurons in the brainstem, and no other misplaced neurons, were marked (antibodies specificity check).

#### Maps of NA-Axons

The DβH maps were built from sections of *M. nemestrina* and *M. mulatta*, whereas the NET maps were prepared from sections of *M. mulatta.* Black and white high-resolution maps of DβH-immunoreactive (-ir) and NET-ir axons were generated as a mosaic of 20x-objective magnification pictures using Neurolucida software (MBF Bioscience, USA) on a personal computer connected to a Zeiss Axioskop microscope (Zeiss, Germany) through a CX9000 camera (MBF Bioscience, USA) and a motorized microscope stage (MBF Bioscience, USA; Heidenhain Corporation, USA).

**Figure 1:**
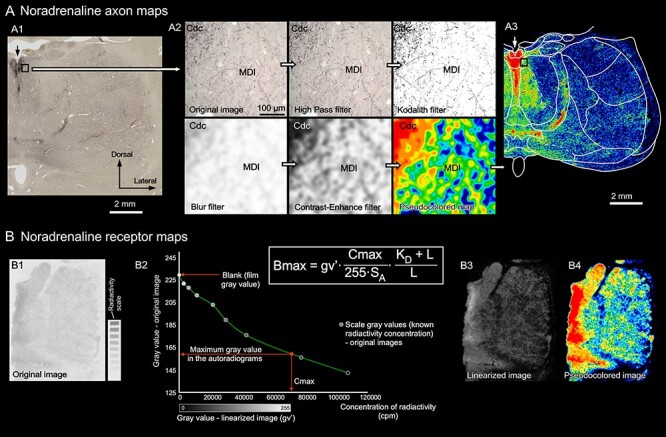
Schematic illustrations of the methods followed to generate the NA axon maps (*A*) and the adrenoreceptor maps (*B*). *A*) Example of the filters applied to obtain the axon maps from a DβH immunostained section from *M. nemestrina.* A1) Low magnification image of the real immunohistochemistry section. A2) 20x-objective magnification microscopic field picture from the field framed in image A1, and images of the sequence of filters that lead to the black and white map (Kodalith filter) and to the pseudocolored map. The calibration bar applies to all the pictures in A2. A3) Complete pseudocolored map from the section in image A1, after applying all filters. In A1 and A3 the arrow points to the midline. *B*) Receptor autoradiographs processing to obtain receptor concentrations and pseudocolored maps: the original autoradiograph and radioactivity scale obtained from the same film (B1) were used to compute a calibration curve (B2). This curve was used to transform the gray value of each pixel in the original image to gray values that are a linear function of the receptor concentration; thus, a linearized image is obtained (B3). The receptor concentration in a specific area is determined from the linearized image using densitometric analysis and the formula in the Figure. Linearized images are transformed into more intuitive pseudocolored images solely for visualization purposes (B4). Abbreviations in B2: B_max_, receptor concentration in the area of interest (fmol per mg of protein: fmol/mgP); C_max_, calculated maximum concentration of radioactivity in the autoradiograph; cpm, counts per minute; gv′, mean gray value of the area of interest in the linearized image; K_D_, equilibrium dissociation constant of ligand-binding kinetics; L, incubation concentration of ligand; S_A_, specific activity of the ligand. For abbreviations in A, see list of Abbreviations.

The “High Pass” filter from Neurolucida was initially used to enhance the axons-background contrast in each 20x-objective magnification picture. Afterward, the Kodalith filter was applied to transform the pictures into monochromatic images, in which black pixels represent the immunostained axons and white pixels represent the nonimmunostained background (Fig. 1*A*). The Kodalith filter threshold was adjusted in each nucleus to achieve the most accurate representation possible; this adjustment was needed because the intensity of the immunostained axons and the background shade varied in the different thalamic nuclei and with the 2 antibodies used. The thresholds were chosen so that maps were comparable with the tissue pictures in terms of axon distribution (see Fig. 3 for comparison of the maps with microphotographs from different thalamic nuclei immunostained with the 2 antibodies used). A monochromatic “.tiff” image was exported from the Neurolucida software with 2 pixel/μm resolution. This image was thoroughly compared with the original pictures to detect artifacts. All artifacts, as well as pixels derived from background, were manually deleted from the monochromatic “.tiff” image using CanvasX software (Canvas GFX Inc., USA). Black pixels representing the axons were transformed to green to differentiate them from the nuclei borders. The green maps shown in this article are newly saved images with a lower resolution than the original maps, due to the large size of the latter.

To create more intuitive images, also comparable with the pseudocolored receptor maps, we transformed the high-resolution DβH monochromatic black and white maps into pseudocolored maps. Using CanvasX software, a Gaussian Blur filter was applied to the black and white maps to generate grayscale maps based on the axons; next, gray levels were adjusted to increase the contrast (same values were applied to all rostrocaudal coronal levels). Grayscale maps were transformed into pseudocolored maps with ImageJ software (National Institutes of Health, USA), using a self-created color scale (Fig. 1*A*).

Sections adjacent to the immunostained ones, and processed for AChE, cresyl-violet, or myelin staining, were used to trace the borders of the thalamic nuclei. The ventricles and blood vessels served as reference marks to transfer the delineated nuclei to the maps. The nuclei were identified following Olszewski (Olszewski 1952) with some modifications previously described by our group (Cavada et al. 1995; Rico and Cavada 1998). When analyzing the receptors (see below), a novel subdivision of the ventral anterior nucleus (VA) was added: VAmcd. This subdivision was characterized as a band of quite big cells, similar to those in magnocellular VA (VAmc), and was only clearly distinct from the latter after analyzing the receptors, in which this region has a consistent and characteristic pattern, different from VAmc and also from parvocellular VA (VApc). We named this subdivision “ventral anterior nucleus, dorsal magnocellular” (VAmcd) (Fig. *6A*,*A*′,*A*″).

#### Optical Densities of NA Axon Maps

To achieve semiquantitative data about the axon densities of the different nuclei, ImageJ software was used to measure the gray value of each nucleus from the Kodalith black and white “.tiff” images of the DβH-ir axonal maps from *M. nemestrina* (levels shown in Figs 4 and 5), with a resolution of 360 pixels per mm of tissue. Since completely white images (no axons) yield a gray value of 255 and completely black images yield a gray value of 0, we calculated the optical densities (OD) as the inverse of those gray values (OD = 255—gray value) to achieve measurements that become higher as the density of axons increase.

ODs for each nucleus in each rostrocaudal level were measured in the 2 hemispheres (left and right), and the final value was calculated as the pondered mean of all the measurements (pondered by area). No evident differences were found between measurements from the left and the right hemispheres.

From our point of view, this is a better approach than measuring the OD from the original histological preparation, since the background in the different nuclei of the thalamus may be quite different, and it would alter the OD measurements.

#### Morphology of NA-Axons

We inspected the DβH immunostained sections with a ×100 oil immersion objective to observe in detail the morphologies of the DβH-ir axons. We then superimposed the delineation of the nuclei and inspected the preparations with a lower magnification objective (×40) to describe the general distribution of the various morphologies on the thalamic nuclei. We describe NA axonal morphology based on DβH-ir axons because in NET-ir axons fine morphologic details were more difficult to distinguish, due to the lower contrast in NET immunohistochemistry compared with that of DβH immunohistochemistry. The diameters of axons and varicosities were assessed with the Neurolucida software measurement tool using the ×100 oil immersion objective. Measurements were made in the axis perpendicular to the direction of the axon, in random high magnification fields of numerous nuclei. Pictures from different axonal morphologies in a variety of nuclei were taken using the ×100 objective (Fig. 5*F*).

### Adrenoceptor Maps

#### Tissue Processing

Receptor autoradiography maps were obtained from 4 hemispheres coming from 3 adult *Macaca fascicularis* monkeys, (6 ± 1 years of age, males). All procedures with the animals followed the European guidelines (86/609/EEC and 2003/65/EC European Council Directives).

A lethal injection of intravenous sodium pentobarbital was administered to each animal. Unfixed brains were immediately removed from the skull. The hemispheres were separated, cut into blocks at the level of the central sulcus, and frozen in isopentane at −40°C. Frozen tissue was stored at −80°C until sectioning.

Serial sectioning was performed on a cryostat (−20°C) in the coronal plane, to obtain 20 μm sections covering the whole hemisphere. Sections were thaw-mounted on gelatin-coated slides and freeze-dried overnight at 500 Torr and −20°C.

#### Ligand Incubation

Sections containing the thalamus from the 3 adult *M. fascicularis* were processed according to established protocols for receptor autoradiography (Zilles et al. 1988; Zilles and Schleicher 1995; Zilles et al. 2002b; Impieri et al*.* 2019) of 2 different adrenoceptors, Alpha-1 and Alpha-2. This procedure involved 3 steps, in brief: 1) preincubation to remove endogenous ligand and rehydration of the tissue; 2) main incubation of adjacent sections with tritiated ligands (to assess total binding) and coincubation of some sections with the tritiated ligand and excess of the Phentolamine Mesylate displacer (Biotrend, Germany ref: BG0426), to evaluate nonspecific binding; and 3) rinsing to eliminate unbound radioactive ligand from the sections. The 2 labeled ligands used in the main incubation were [^3^H]-prazosin (Perkin-Elmer, Germany, ref: NET 823), which binds mainly to the Alpha-1 receptors, (Hornung et al. 1979; Cash et al. 1985; Zilles et al. 2002a), and [^3^H]-UK-14,304 (5-Bromo-N-(4,5-dihydro-1H-imidazol-2-yl)-6-quinoxalinamine) (Perkin-Elmer, Germany, ref: NET 853), the high-affinity Alpha-2 ligand (Cambridge 1981; Loftus et al. 1984). See Table 1 for the incubation conditions in each step.

**Table 1:** Incubation conditions and ligand specifications for receptor autoradiography

Receptor	Alpha-1	Alpha-2
[^3^H]-Ligand	[^3^H]-Prazosin	[^3^H]-UK-14,304
Pharmacology	Antagonist	Agonist
Ligand concentration (L)	0.2 nM	0.64 nM
K_D_ value	0.2 nM	1.4 nM
Specific activity S_A_	80.5 Ci/mmol	81.2 Ci/mmol
Displacer	Phentolamine mesylate	Phentolamine mesylate
Displacer concentration	10 μM	10 μM
Incubation buffer	50 mM Na/K-phosphate buffer (pH 7.4)	50 mM Tris-HCl + 100 μM MnCl2 (pH 7.7)
Preincubation	15 min, 22°C	15 min, 22°C
Main incubation	60 min, 22°C	90 min, 22°C
Rinsing	1) 2 × 5 min, 4°C 2) distilled water, 1 × 22°C	1) 5 min, 4°C 2) distilled water, 1 × 22°C

Nonspecific binding is only taken into consideration when it amounts to more than 10% of the total binding sites marked by the ligand, this was not the case in any of the studied brains.

Adjacent series of sections were processed with a modified silver method (Merker 1983) that produces Nissl-like images, or myelin silver stained (Gallyas 1979).

#### Autoradiography, Image Acquisition, and Generation of Receptor Distribution Maps

Radioactively labeled sections were coexposed with standards of known radioactivity concentrations (RPA501, Microscales, Amersham, Germany) at 4°C against [^3^H]-sensitive film (Hyperfilm, Amersham, Germany) for 6 weeks. The films were developed with Kodak D19 developer for 5 min and fixed with Kodak Unifix for 10 min at room temperature.

The resulting autoradiographs were subsequently processed by densitometry and transformed into “linearized images” in which the gray values are a linear function of the concentration of radioactivity following previously described methodology (Zilles et al. 2002b; Palomero-Gallagher and Zilles 2018).

Pseudocolored images were produced from the linearized image by assigning 11 colors in a spectral sequence to equally spaced gray value ranges using Matlab software, so that each color is associated with a concentration of radioactivity or concentration of receptor in fmol per mg of protein (fmol/mgP), and this association is also displayed on a color-scale bar.

The thalamic nuclei were examined on adjacent Nissl and myelin stained sections using a table projector and high-resolution digitalized images from the tissue. Then, the nuclear borders were drawn onto low-resolution pictures from Nissl and myelin stained sections and transferred to the pseudocolored images using thalamic and nonthalamic anatomical landmarks.

#### Quantification of the Receptor Concentrations in Thalamic Nuclei

Using the above-defined borders of the thalamic nuclei, the mean gray value of each delineated area in the linearized images was obtained using an in-house-developed software. The concentration of receptors for each nucleus in each section was calculated according to the following equation:

$$ {\mathrm{B}}_{\mathrm{max}}={\mathrm{gv}}^{\prime}\times \frac{{\mathrm{c}}_{\mathrm{max}}}{255\times{\mathrm{S}}_{\mathrm{A}}}\times \frac{{\mathrm{K}}_{\mathrm{D}}+\mathrm{L}}{\mathrm{L}} $$

Where B_max_ is the receptor concentration in the area of interest in fmol/mgP, gv’ is the mean gray value of that area in the linearized image, C_max_ is the calculated maximum concentration of radioactivity in the autoradiograph, K_D_ is the equilibrium dissociation constant of ligand-binding kinetics, L is the incubation concentration of ligand, and S_A_ is the specific activity of the ligand (for the values of S_A_, K_D_, and L, see Table 1).

A mean receptor concentration for each nucleus in each hemisphere was calculated by pondering the concentration value of the nucleus in the sections where it was present by the area of the nucleus (number of pixels) in those sections. Afterward, an arithmetic mean from the 4 hemispheres was calculated to obtain the uncorrected receptor concentration of each nucleus (Table 3).

#### Quenching Correction

When using tritiated ligands in quantitative receptor autoradiography to study receptor distribution patterns within a nonhomogenous structure, differential quenching of beta-emission may strongly affect the apparent distribution of radioactivity (Zilles et al. 1990), and therefore the apparent receptor distribution. According to Zilles et al. 1990, quenching might be a cause of “mismatch” between presynaptic markers and receptors distributions.

To avoid misinterpretation of the data, a thorough quenching correction was made here, based on the uniform binding of ^3^H-succinimidyl-propionate to CNS tissue sections (Herkenham and Sokoloff 1984): in sections from 3 of the 4 hemispheres used for receptor autoradiography, a [^3^H]-succinimidyl-propionate autoradiography was performed (see Table 2 for the incubation conditions).

**Table 2:** Incubation conditions for the succinimidyl binding assay

[^3^H]-Ligand	[^3^H]-Succinimidyl-propionate
Ligand concentration	4 nM
Incubation conditions	50 mM Tris-HCl buffer (pH 7.2), 30 min, 22°C
Rinsing	3 × 50 mM Tris-HCl buffer (pH 7.2), 4°C

Autoradiographs from the succinimidyl binding assays were processed to obtain linearized images and “succinimidyl concentrations” in each section the same way as for the receptor autoradiographs. The mean “succinimidyl-concentration” (Bmax_SUCC_) was then calculated for each nucleus from the sections of 3 hemispheres processed for succinimidyl binding. Since succinimidyl binds uniformly to the whole section, all the concentration differences obtained from the autoradiographs were due to the quenching effect, and therefore the quenching (%) in each nucleus can be estimated. This correction can be then applied to the uncorrected receptor concentration.

All brain regions have some quenching effect, and therefore we have no access to the real succinimidyl propionate concentration, which would be obtained from a region with 0% quenching, or in other words, a region with 100% autoradiographic efficiency, RE [RE = 100—Quenching, (Zilles et al. 1990)]. We calculated the succinimidyl propionate concentration associated with 100% RE indirectly from white matter succinimidyl-propionate concentration: it has been proved that white matter has a 40% RE (Zilles et al. 1990). Therefore, we measured the succinimidyl-propionate concentration in the white matter (corona radiata superior to the temporal sulcus) of every coronal level in the 3 hemispheres to obtain the mean white matter “succinimidyl concentration” (Bmax_WM_). This Bmax_WM_ value corresponds to 40% RE, so the “theoretical” 100% RE succinimidyl-propionate concentration value can be calculated. After this inference, all the REs for each nucleus were calculated as a percentage of the 100% concentration obtained before:

$$ \begin{align*} & {\mathrm{RE}}_{\mathrm{in}\ \mathrm{a}\ \mathrm{defined}\ \mathrm{brain}\ \mathrm{region}}\\ & \quad=\tiny\frac{\mathrm{Succinimidyl}\ \mathrm{Bmax}\ \mathrm{in}\ \mathrm{a}\ \mathrm{defined}\ \mathrm{brain}\ \mathrm{region}\times 100}{\frac{{\mathrm{Succinimidyl}\ \mathrm{Bmax}}_{\mathrm{WM}}\times 100}{40}} \end{align*}$$

We then calculated the corrected receptor densities for each nucleus (Table 3) as follows:

$$ \mathrm{Corrected}\ \mathrm{receptor}\ \mathrm{density}=\frac{\mathrm{Uncorrected}\ \mathrm{receptor}\ \mathrm{density}\times 100}{\mathrm{RE}} $$

For those rare nuclei or subnuclei where no succinimidyl OD were available [area X, medial geniculate nucleus parvocellular portion {GMpc}, posterior nucleus {Po}, inferior pulvinar nucleus {Pul I}, and paraventricular {Pv}], RE was calculated as the mean RE of selected nuclei with similar myelin densities.

#### Alpha-1/Alpha-2 Relative Ratio

We considered it interesting to analyze whether Alpha-1 or Alpha-2 receptors predominate in a particular nucleus. Alpha-1 receptor concentrations are higher in almost all the thalamic nuclei with only 2 exceptions. However, we noted that the increase or decrease of Alpha-1 and Alpha-2 receptors was not homogenous: for example, in some nuclei, even if the Alpha-2 receptor concentration was lower than that of Alpha-1, Alpha-2 concentration was higher than its mean in the thalamus, whereas Alpha-1 concentration was around its own mean, or even lower.

To illustrate differences in the relative concentrations of Alpha-1 and Alpha-2 receptors, we used a “relative ratio”: we first calculated a relative concentration for each receptor in each nucleus, by dividing the receptor concentration in the nucleus by the mean receptor concentration in the thalamus. If this relative concentration is above 1, this nucleus shows a higher receptor density than its mean in the thalamus, whereas when it is lower than 1, the receptor concentration in this nucleus is below its mean. Then, we divided the Alpha-1 relative concentration by the Alpha-2 relative concentration, to obtain the Alpha-1/Alpha-2 “relative ratio” (Table 3). If this ratio is above 1, Alpha-1 receptor concentration compared with its own mean is higher than that of Alpha-2 compared with its own mean. The opposite would be true if the ratio is below 1.

#### Statistical Analysis

We used the SPSS software (IBM, USA) to check the normal distribution of the variables “DβH OD,” “Alpha-1 concentration,” “Alpha-2 concentration,” and “total Alpha receptor concentration” by means of the Kolmogorov–Smirnov test. All the variables had normal distributions except for DβH OD. We then performed the nonparametric Spearman’s rho to assess the correlation between variables, because we wanted to correlate receptor concentrations with DβH OD values, and this variable follows a nonnormal distribution.

Data from 60 nuclei and subnuclei were analyzed. Values for 51 nuclei were available for the 4 studied variables, 3 were available only for DβH OD [densocellular anteromedial nucleus {AMdc}, ventral sector of mediodorsal nucleus {MDv}, and zona incerta {ZI}] and 6 were available only for the Alpha receptor concentrations [central superior {Cs}, magnocellular lateral geniculate {GLmc}, parvocellular lateral geniculate {GLpc}, posterodorsal medial geniculate {GMpd}, mediodorsal nucleus pars multiformis {MDmf}, and VAmcd]. Data from tracts [e.g., mamillothalamic tract {*Tmt*}, corticotectal tract {*Ct*}, or external medullary lamina {*Lme*}] were not considered.

Graphs in Figure 7 were created using Excel (Microsoft, USA) and were edited with CanvasX software.

## Results

### Antibodies Specificity: Control Experiments

**Figure 2:**
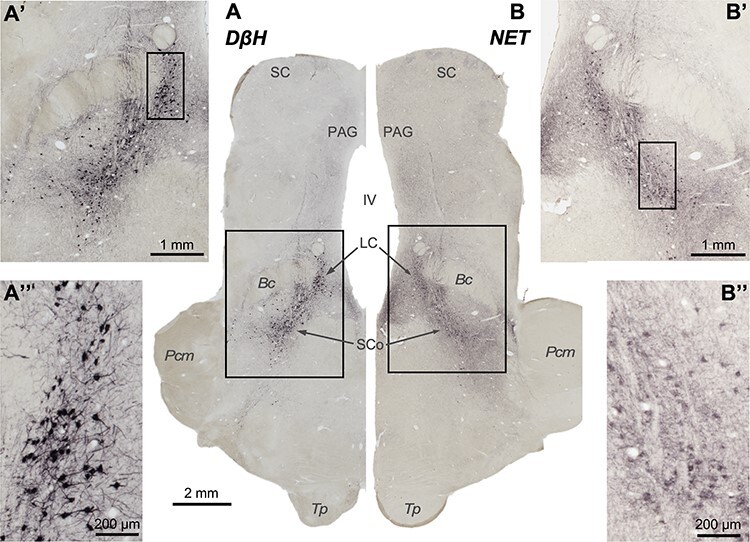
Pictures from brainstem slices through the coronal plane, immunoprocessed to reveal the 2 markers used in this work: DβH (*A*, *A*′, and *A*″) and NET (*B*, *B*′, and *B*″). Rectangles represent fields shown at higher magnification. Note that putative noradrenergic neurons are stained in the locations where NA neurons have been previously described, and not elsewhere. The contrast is higher in the neuronal bodies immunostained for DβH than in the neuronal bodies immunostained for NET, possibly due to the characteristics and cellular location of the markers. Importantly, both markers show selectivity for NA cells.

The 2 antibodies used stained groups of neuronal somas of putative noradrenergic cells in the brainstem, where NA cells are located (Fig. 2). The immunohistochemistry from the series covering the whole brain was inspected: no neuronal body was found anywhere rostral to the pons.

The 2 antibodies revealed well-established axonal fields in regions like the neocortex and the hippocampus.

Negative control experiments, where sections had been processed omitting the primary antibodies, yielded no specific immunostaining.

### NA Axon Maps

#### Comparison between NET-ir and DβH-ir Axons Distributions

**Figure 3:**
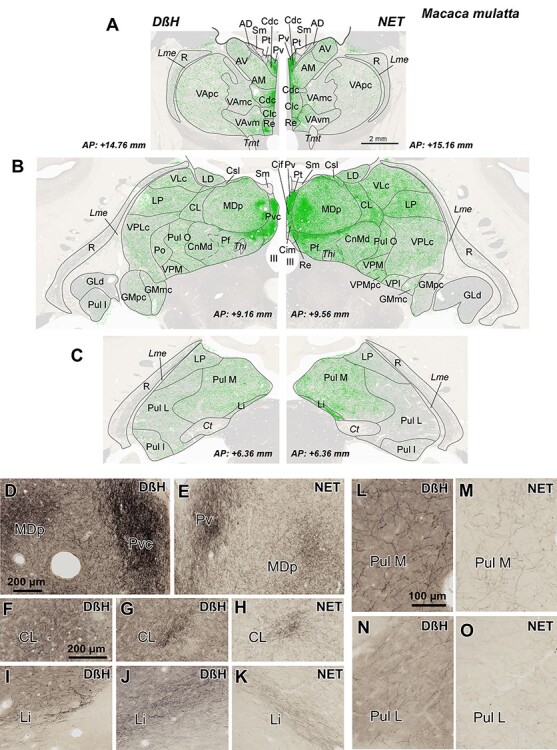
*A*–*C*) Distribution of NA axons (green) in 3 representative coronal sections of the *M. mulatta* thalamus, revealed by DβH (left) and NET (right) immunostaining*.* Note that the distribution patterns are very similar, independently of the marker used: the midline nuclei, the intralaminar nuclei (particularly Pf nucleus), and the medial sector of MD are the most innervated regions. NA axons are represented over attenuated photographs of adjacent sections stained for AChE, which were used to identify the nuclear borders. The stereotaxic anteroposterior (AP) level is indicated for each section. *D*–*O*) Pictures from immunostained sections allowing comparison between DβH and NET immunohistochemistry. Note that DβH immunostaining gives a higher contrast, but the distribution is similar for both markers. *D*,*E*) Dense patches present in Pv and MDp; microscopic fields from maps in B. *F*–*H*) Dense innervation in CL nucleus taken from the DβH map in B (F), from NET map in B (H), and from a section in a slightly different rostrocaudal level from the same animal, where DβH immunostaining in CL is more comparable to the one shown for NET immunostaining in B and H (G). *I*–*K*) Labeled-ir axons in Li nucleus taken from the DβH map in C (I), from NET map in C (K) and from a DβH immunostained section in a different animal, at a rostrocaudal level comparable to that of K (J). Note that, in I, as in C (left), the NA innervation of Li nucleus is near the posterior end of the nucleus, whereas the density patterns in C (right), J, and K are comparable between the 2 markers. Pictures of Li in I-K are shown as specular images for DβH and NET immunostaining to facilitate comparison with the maps in C. *L*–*O*) Microphotographs from Pul M and Pul L showing a higher innervation in Pul M. The calibration bars are common for *A*-C, D-E, F-K, and L-O. See the list of Abbreviations for identification of each thalamic nucleus.

**Figure 4:**
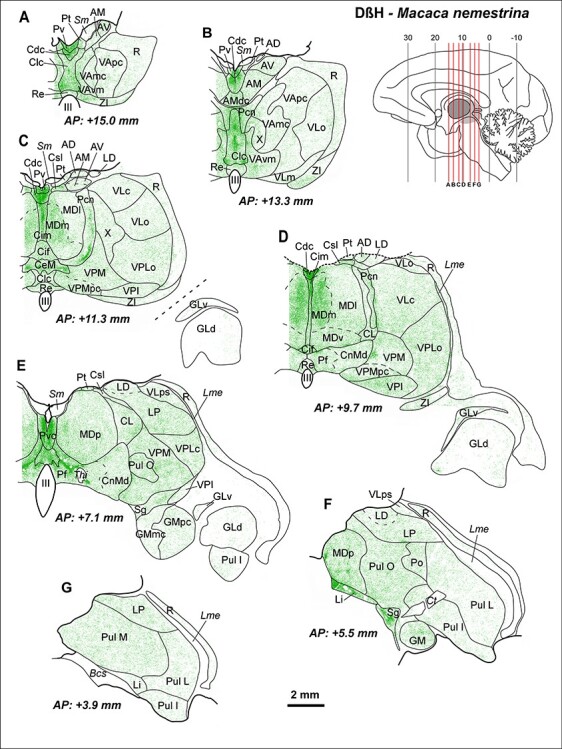
Maps from coronal sections of *M. nemestrina* depicting the distribution of NA axons (green) revealed by DβH immunohistochemistry, in representative stereotaxic AP levels. In the upper right corner, there is a scheme of a medial sagittal view of a hemisphere, modified from Olszewski (1952), where the thalamus is highlighted in gray, and red lines represent the AP levels shown in the maps. DβH-ir axons are present in all thalamic nuclei, but highly heterogeneously distributed, following a pattern similar to the one present in *M. mulatta* (Fig. 3). Note the dense innervation in midline nuclei (*A*–*E*), intralaminar nuclei (*C*–*E*) (particularly Pcn and Pf), Li, Sg (*F*), and in MDm (*C*,*D*). A moderate innervation is present in ventral motor nuclei, with higher densities in VAvm and X (*A*–*C*). A very low innervation is present in the GLd nucleus (*C*–*E*), where only some axons are identifiable. A moderate innervation is present in the somatosensory relay nuclei VPM and VPLc (*C*–*E*), as well as in the auditory relay nucleus GM (*E*,*F*). A heterogeneous innervation is present in the pulvinar complex (*E*–*G*). In R, NA axons are present mostly in its anterior and dorsal regions (*A*–*E*); note that ventral R regions in D-G are virtually devoid of NA innervation. The AP stereotaxic level is indicated for each section, and the calibration bar applies to all the maps in the Figure. See list of Abbreviations for identification of each thalamic nucleus.

Both DβH-ir and NET-ir axons were present in all thalamic nuclei, albeit with notable density differences, described in the next chapter. Figure 3*A*–*C* shows that the 2 NA phenotypic markers gave similar distribution patterns. Some disparities appreciated in the maps (e.g., CL, Li, Pul L, and Pul M) are due to slight differences in the rostrocaudal level mapped in for each marker and not to the marker itself. Figure 3*D*–*O* shows pairs of pictures from several nuclei at similar rostrocaudal levels immunostained for DβH and NET. Note that axon density patterns are similar. However, the contrast was better in the DβH immunohistochemistry than in the NET immunohistochemistry (Figs 2 and 3*D–O*). Therefore, we mapped systematically the NA-axonal distribution in coronal levels from the DβH immunostained sections (Fig. 4).

As expected, the overall distribution patterns of NET-ir and DβH-ir axons were highly coincident in the 2 studied species, *M. mulatta* and *M. nemestrina* (Figs 3 and 4).

#### Distributions of DβH-ir Axons

**Figure 5:**
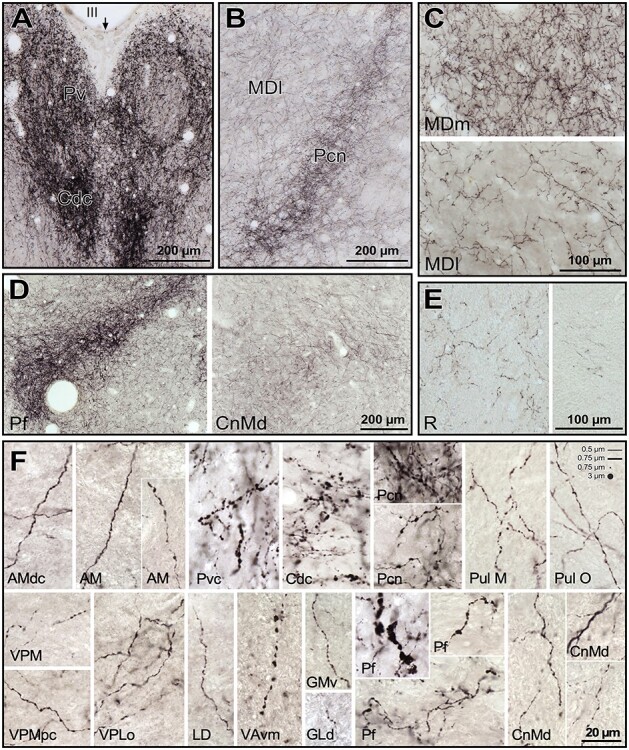
Microphotographs from histological sections of *M. nemestrina*, processed for DβH immunohistochemistry, from which the maps in Fig. 4 were obtained. *A*) Midline nuclei, mainly Pv and a portion of Cdc in the lower part. The arrow points to the midline. *B*) Anterior intralaminar nucleus Pcn, and rostral part of MDl. Note in *A*) and *B*) the dense innervation in Pv, Cdc and Pcn even at low magnification. *A*) and *B*) were taken from the histological section corresponding to the map in Fig. 4*C*. *C***)** Comparison of the NA axon densities in 2 different regions of MD, with MDm having a denser innervation than MDl (pictures taken from section in Fig. 4*D*). *D*) Low magnification pictures of the posterior intralaminar CnMd-Pf complex, where the dense innervation in posterior Pf is in contrast with the moderate innervation in CnMd (from section in Fig. 4*E*). *E*) Microphotographs of R in 2 different fields. The one on the left is taken from a dorsal region of the section in Fig 4*D*; and the one on the right from a ventral region of the section in Fig 4*F*. *F*) High magnification pictures from a variety of nuclei, where different axonal morphologies are represented. Note the general varicose morphology, with the thicker varicosities present in midline nuclei (Pvc, Cdc), Pf, and VAvm. In general, intervaricose segments were thin, with calibers ranging from 0.5 to 0.75 μm; sometimes the intervaricose regions of the axons were unidentifiable (e.g., VAvm). Varicosities ranged from 0.5 to 3 μm. In the upper right corner, scaled lines and dots with the mentioned calibers are depicted, for comparison with the microphotographs. See list of Abbreviations for identification of each thalamic nucleus.

The most innervated thalamic nuclei were the midline nuclei (Figs 3*A*–*B*, 4*A*–*E*, and 5*A*). High NA axon densities were also present in some intralaminar nuclei, including the paracentral nucleus (Pcn) (Figs 4*C*–*D* and 5*B*) and the centromedian-parafascicular complex, where the densest innervation was in the parafascicular nucleus (Pf), particularly at caudal levels (Figs 3*B*, 4*D*–*E*, and 5*D*).

Besides the midline and the Pf nuclei, the most innervated region was the medial sector of the mediodorsal nucleus (mediodorsal nucleus: MD; medial sector: MDm) (Figs 4*C*–*D* and 5*C*). Within the anterior group, DβH-ir axons were in general present in moderate densities, but also heterogeneously distributed: the anterodorsal nucleus (AD) held a low density of DβH-ir axons whereas the anteromedial (AM) and anteroventral (AV) nuclei exhibited moderate densities, with the densest innervation in AMdc (Figs 3A and 4*A*–*D*). The adjacent lateral dorsal nucleus (LD) was also moderately innervated, as well as the lateral posterior nucleus (LP) (Figs 3 and 4*D*–*G*). The ventral motor nuclei [VA and ventral lateral {VL}] exhibited a moderate, and to some extent, heterogeneous NA innervation (Figs 3*A*–*B* and 4*A*–*D*), with the ventromedial subdivision of the VA nucleus (VAvm) and area X having the highest innervation among these nuclei (Fig. 4*B*). Somesthetic and auditory relay nuclei [ventral posteromedial {VPM}, caudal ventral posterolateral {VPLc}, and medial geniculate {GM}] showed a moderate density of DβH-ir axons (Figs 3*B* and 4*C*–*F*). The visual relay nucleus, GLd, held the lowest NA innervation in the thalamus (Figs 3*B* and 4*C*–*E*). Within the pulvinar group, the oral pulvinar (Pul O) nucleus displayed the highest DβH-ir innervation, medial pulvinar (Pul M) nucleus exhibited moderate densities of DβH-ir axons, whereas the lateral pulvinar (Pul L) and Pul I held rather low densities (Figs 3*B*–*C* and 4*E*–*G*). The reticular nucleus (R) showed an overall low density of DβH-ir axons (Figs 3–5*E*). Most DβH-ir axons in R were present in its anterior and dorsal regions (Figs 3–5*E*). Further details on the distribution of DβH-ir axons are in Figures 3 and 4.

#### Morphology of DβH-ir Axons

Almost all the DβH-ir axons in the thalamus were varicose; both the axons and the varicosities had diverse sizes (Fig. 5*F*). Thin axonal profiles (diameter ≤ 0.5 μm) were more frequent than thick ones (diameter > 0.5 μm).

Great variation was observed in the size and shape of the varicosities. Their diameters ranged between 0.5 and 3 μm. Depending on their morphology, axonal profiles were divided into spherical varicose axons and pleomorphic (irregularly shaped) varicose axons. In general, spherical varicose axons were thin (the intervaricose segments of the axons were sometimes indistinguishable, Fig. 5*F*, e.g., VPM, PulM), whereas pleomorphic varicose axons were both thin and thick.

In some nuclei [midline, Pcn, Pf, VAvm, nucleus limitans {Li}, suprageniculate nucleus {Sg}], spherical varicose axons were more abundant than in the remaining nuclei. It is also in those nuclei, which are densely innervated by NA, where the thicker varicosities are present (Fig. 5*F*) Likewise, occasional atypical “swollen” axons were observed, mostly in midline, Pf, Sg, and Li nuclei (Fig 5*F*, Pf). These “swollen” axons appear similar to dopamine “swollen” axons described in the primate thalamus (Garcia-Cabezas et al. 2007). MDm, despite its dense NA innervation, does not present axons with the thickest varicosities, like those of midline nuclei. The majority of DβH-ir axons in the nuclei with moderate or low innervation was pleomorphic and thin, although spherical varicose axons were observed randomly in many of them.

### Adrenoceptor Maps

#### Alpha-1 and Alpha-2 Adrenergic Receptor Distribution

Densities and distributions of Alpha-1 and Alpha-2 receptors were considerably heterogeneous throughout the thalamic nuclei. Concentrations of Alpha-1 receptors were in general higher than those of Alpha-2 receptors (Table 3).

**Figure 6:**
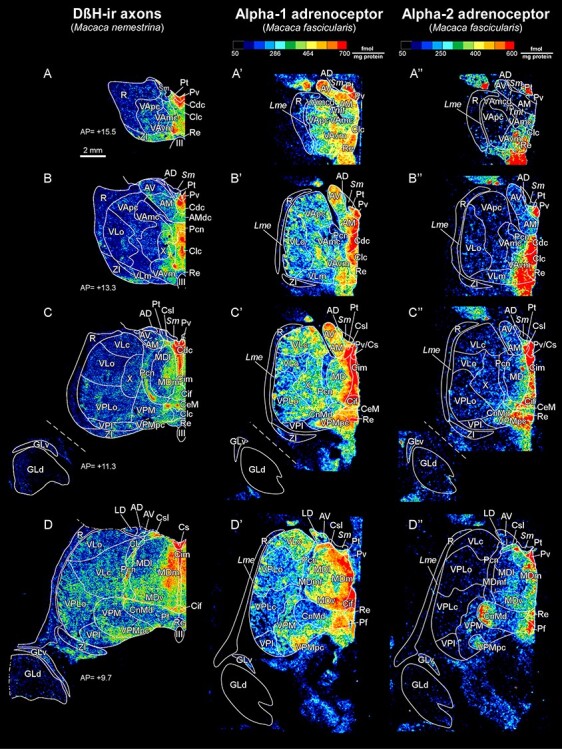

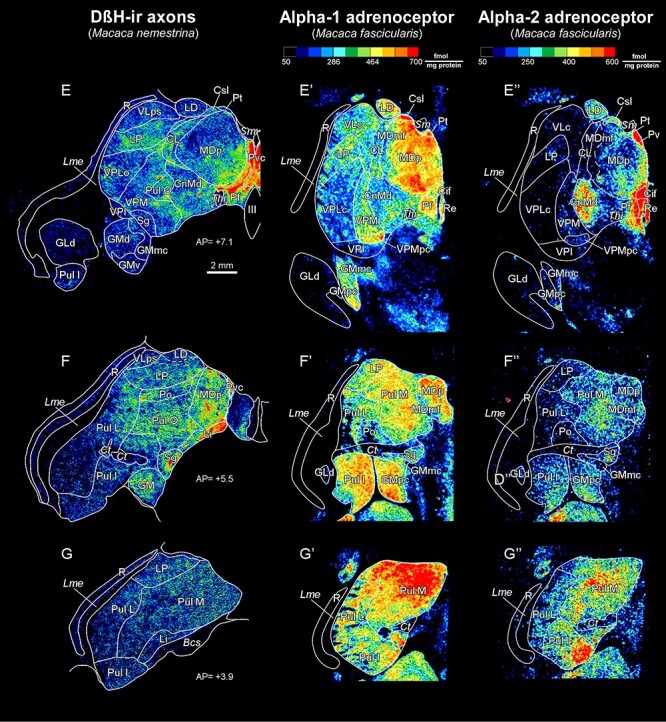
Comparison between the pseudocolored maps of the DβH-ir axons (*A*–*G*), Alpha-1 (*A*′-G′), and Alpha-2 (*A*″–G″) receptor distributions. Note that, in general, densities of Alpha-1 receptors are higher than those of Alpha-2 receptors, and both together match the axonal distribution, with some exceptions: Pcn (*C*–*D*; *C*′–D′; *C*″–*D*″), CnMd (*D*–*E*, *D*′–*E*′, *D*″–*E*″), Pul M (*G*, *F*′–*G*′, *F*″–*G*″), and Pul I (*F*–*G*, *F*′*–G*′, *F*″–*G*″). The calibration bar applies to all the maps in this Figure. See list of Abbreviations for identification of each thalamic nucleus.

The highest Alpha-1 densities were located in the midline nuclei (Fig. 6*A*′–D′), some regions of MD (Fig. 6*D*′–*E*′), the parvocellular part of VPM (VPMpc) (Fig. 6*C*′–*E*′), Pul M (Fig. 6*F*′–*G*′), GM (Fig. 6*F*′), anterior regions of Pul I (Fig. 6*F*′), and the anterior group, particularly AV and AM (Fig. 6*A*−*C*′). The ventral motor nuclei showed a moderate density of Alpha-1 adrenergic receptors, with higher concentrations in VAvm, VAmcd, area X, and ventral lateral nucleus, caudal part (VLc) (Fig. 6*A*′–*D*′). The nuclei with the lowest Alpha-1 receptor densities were the GLd (Figs 6*C*′–*F*′) and R (Fig. 6*A*′–*G*′). Alpha-1 receptors in the intralaminar nuclei were rather low, except for Pf (Fig. 6*D*′–*E*′), which exhibited a high Alpha-1 receptor density.

Alpha-2 receptor densities were lower than Alpha-1 in all thalamic nuclei, with the exception of the lateral geniculate nucleus (both dorsal and ventral, GLd and GLv) and the posterior intralaminar centromedian nucleus (CnMd) (Fig. 6*C*″–*E*″). CnMd held the highest Alpha-2 receptor density in the thalamus, together with most midline nuclei (Fig. 6*A*″–*E*″). Other nuclei with high Alpha-2 concentrations were the Pf (Fig. 6*D*″*–E*″), GM (Fig. 6*F*″), Pul M (Fig. 6*F*″–*G*″), Pul I (Fig. 6*F*″–*G*″)—particularly its most posterior and medial regions, LD (Fig. 6*E*″), and VAvm (Fig. 6*A*″–*B*″). The lowest Alpha-2 receptor densities were present in R, AD, and the remaining ventral motor nuclei [VA, VL, and ventral posterior lateral nucleus, oral part {VPLo}].

#### Quantification of Alpha-1 and Alpha-2 Receptor Concentrations

Table 3 shows complete quantitative data on Alpha-1 and Alpha-2 adrenoceptors throughout the macaque thalamic nuclei. After quantification and quenching correction, minor changes in the qualitative observations based on the maps were detected. These differences are due to the quenching effect, and also to the fact that a single case is represented in Figure 6, whereas Table 3 provides the mean receptor densities and standard deviations. Area X and VLc turned out to have a relatively higher concentration than shown in the maps. AV was found to have a similar Aplha-1 concentration to AM, whereas in the maps, it seems to have a higher concentration of Alpha-1 receptors than AM.

The mean Alpha-1 receptor concentration from all thalamic nuclei after quenching correction was 739 fmol/mgP, ranging from 1158 fmol/mgP [central nucleus, intermediate part {Cim}] to 205–207 fmol/mgP (GLd and R). The mean Alpha-2 receptor concentration from all thalamic nuclei was 388 fmol/mgP, ranging from 982 fmol/mgP [caudal paraventricular nucleus {Pvc}] to 151–155 fmol/mgP (AD and R).

**Figure 7:**
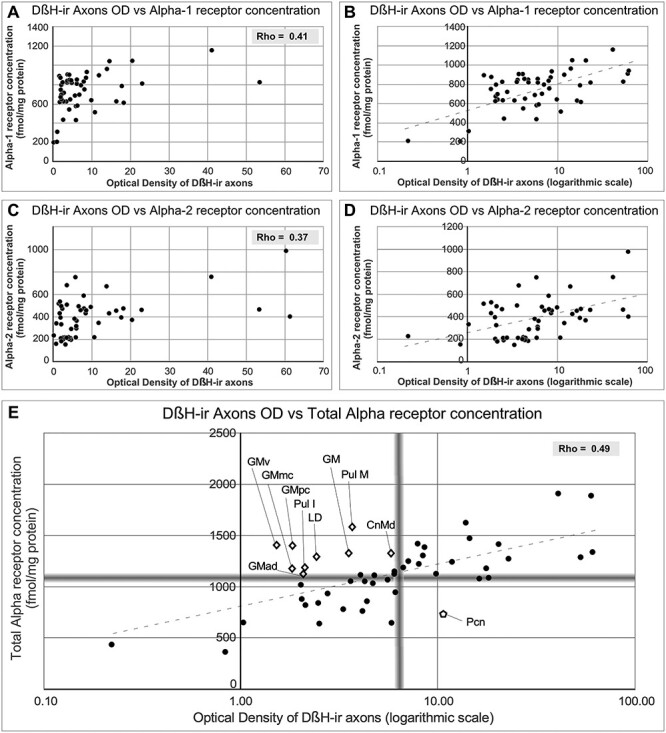
Graphs representing the correlations between Alpha receptor concentrations and OD of DβH-ir axons. *A*) and *C*) Representation of the values of Alpha-1 receptor concentration (*A*) and Alpha-2 receptor concentration (*C*) vs. the DβH OD. Rho value is given on each graph. *B*) and *D*) Transformation of DβH OD to logarithmic scale changes the distribution of the data to a linear pattern. *E*) Representation of values of total Alpha receptor concentrations vs. OD of DβH-ir axons (transformed to logarithmic scale). Blurred gray lines represent the median value in each axis. Note that after transformation, receptors and axons follow a linear correlation, and nuclei in which axon and receptor densities are not completely coincident can be identified in the graph: open diamonds represent nuclei containing high Alpha receptor densities and low DβH-ir axon densities. The open pentagon represents the Pcn nucleus, which has a high DβH-ir axon density and low Alpha receptor densities.

#### Comparison between NA Axons and Alpha-Adrenergic Receptors

In general, NA axons are more coincident with Alpha-1 receptors than with Alpha-2 receptors. If ODs of DβH-ir axons and total Alpha receptor densities are compared within each nucleus individually (Figs 6 and 7E, Table 3), both matches and mismatches can be identified. Matching distributions of NA axons and Alpha receptors are present in the midline nuclei, MD (particularly in MDm and MDp), Pf, VAvm, and superior lateral part of the central nucleus (Csl), where both receptors and axons are high; or in GLd, R, and Pul L, where both receptors and axons are low. However, in other nuclei, axons and receptors are not so coincident: GM, Pul M, Pul I, LD, and CnMd exhibit moderate NA innervation but comparatively high Alpha receptor densities, either Alpha-1 or Alpha-2, or both. A reverse relationship is present in the anterior intralaminar Pcn, where there is dense NA innervation, whereas Alpha-1 and Alpha-2 receptors are scarce.

After statistical correlation between NA axonal densities (OD data) and Alpha receptor concentrations by means of the nonparametric Spearman’s Rho test, both Alpha-1, Alpha-2, and total Alpha adrenoceptors concentrations show a nonlinear correlation with the axonal densities; which was significant at the 0.01 level (2-tailed) (Rho_Alpha-1 vs. OD_ = 0.41; Rho_Alpha-2 vs. OD_ = 0.37; Rho_Alpha-Total vs. OD_ = 0.49; Rho_Alpha-1 vs. Alpha-2_ = 0.47). The correlation between receptors and axons seems to follow a logarithmic pattern; in fact, using a logarithmic scale in the OD axis the data align (Fig. 7*A*–*D*). These correlations are depicted in Figure 7. Figure 7*E* features the nuclei presenting “nonmatching” densities between axons and receptors: in blurred gray, the median values for the 2 axes establish limits, which generate 4 quadrants: in the upper-right quadrant are the nuclei with high NA-axons and high Alpha receptor densities, in the lower-left quadrant are the nuclei with low density of NA axons and low receptor concentration, in the lower-right quadrant are the nuclei with high NA axonal density and low receptor concentration, and in the upper-left quadrant the nuclei with low axonal density and high receptor density.

#### Analysis of the Data in the Context of Functional Groups Of Nuclei

When functionally related nuclei are arranged together, it is possible to analyze if the distribution of DβH-ir axons and theAlpha-adrenergic receptor distribution follow specific patterns. This analysis is shown in Table 3.

**Table 3:** Values of DβH ODs, and of Alpha-1 and Alpha-2 receptor concentrations before and after quenching correction, total Alpha receptor concentrations, and Alpha-1/Alpha-2 ratios. Colors indicate the position of each value in relation with its own mean. In the columns with color, white represents the percentile 50. Blue and magenta represent values lower and higher, respectively, than percentile 50. The darkest shades represent the extreme values. In the Alpha-1/Alpha-2 ratio column, yellow represents nuclei that have similar relative Alpha-1 and Alpha-2 receptors (Alpha-1/Alpha-2 ratio = 1), orange represents nuclei that have higher relative Alpha-1 concentrations, and green represents nuclei that exhibit higher relative Alpha-2 concentrations

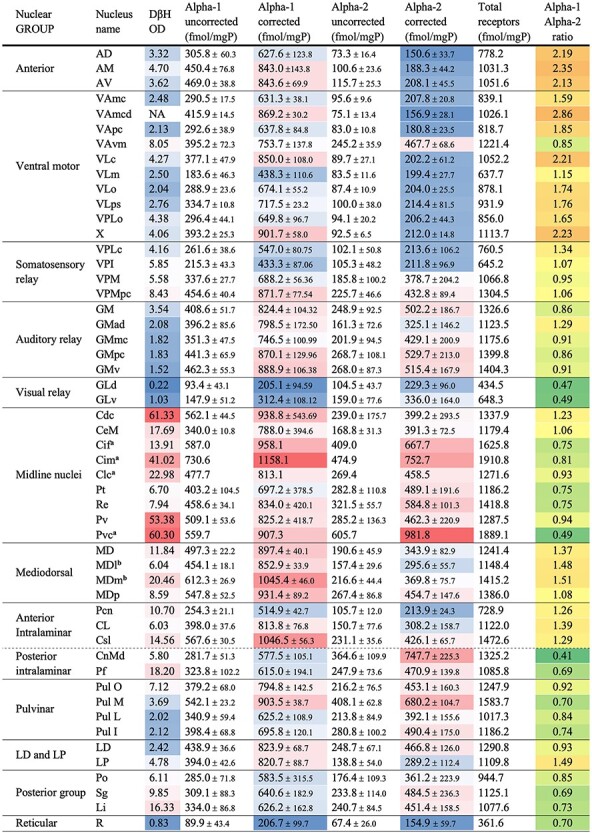

^a^Data from nuclei with this symbol were available only in one hemisphere, and therefore SD is not shown

^b^MDm and MDl borders for measuring Alpha receptor concentrations were not precise, since adjacent sections processed for AChE staining were not available for brains processed for receptor autoradiography.

The anterior group of nuclei and the ventral motor nuclei have a moderate DβH-ir axonal innervation and also a moderate receptor concentration, which tends to be relatively higher for the Alpha-1 than for the Alpha-2 receptors.

The ventral somesthetic relay nuclei have a similar pattern, but axons are a bit more abundant than in the above groups, andreceptors, both Alpha-1 and Alpha-2, tend to be closer to their mean.

The visual relay GLd nucleus has a low DβH-ir axonal innervation, and low Alpha-1 and Alpha-2 receptor concentrations. A similar pattern is present in R. The auditory GM nucleus displays a moderate DβH-ir axonal innervation, and rather high receptor concentrations, both Alpha-1 and Alpha-2. Alpha-2 receptors in GLd, GM, and R nuclei tend to be more above their mean than Alpha-1 receptors.

The midline nuclei show a profuse DβH-ir axonal innervation and have high Alpha-1 and Alpha-2 receptor concentrations, which tend to be more above the mean for the Alpha-2 receptors than for Alpha-1 receptors (see values of Alpha-1/Alpha-2 ratio in Table 3).

MD as a whole shows a moderate-to-high DβH-ir axonal innervation and shows a high Alpha-1 receptor concentration, but around the mean Alpha-2 receptor concentration. When MD subdivisons are considered, it appears that MDm and MDp show more NA axons and higher Alpha-receptor densities than MDl.

The intralaminar nuclei are in general highly innervated, but receptor differences are present between the anterior and the posterior groups: the nuclei in the anterior group tend to show an average concentration of receptors, with the Alpha-1 concentrations slightly above the mean (except in Pcn); in the posterior group, Alpha-1 concentrations are slightly lower than their mean, whereas Alpha-2 concentrations are above their mean.

In the pulvinar nuclei, heterogeneous patterns of innervation and Alpha receptor densities are present: Pul O contains a high density of DβH-ir axons, Pul M a moderate density, and Pul I and Pul L, a low density. Likewise, in the Pul O and Pul M, Alpha receptors are in general higher than in PuL L and Pul I. All 4 nuclei in this group display higher relative concentrations of Alpha-2 receptors than those of Alpha-1.

The posterior group shows a high density of NA axons, rather low Alpha-1 receptor densities, and moderate Alpha-2 receptor densities (and therefore a low Alpha-1/Alpha-2 relative concentrations ratio).

As a general observation, it can be noted that the anterior complex, ventral motor nuclei, anterior intralaminar nuclei, and MD nucleus show a high Alpha-1/Alpha-2 relative ratio, and therefore have relative higher Alpha-1 concentrations, whereas anatomically posterior nuclei (GM, GL, posterior intralaminar, Pulvinar complex, posterior intralaminar group) and midline nulei show a low Alpha-1/Alpha-2 relative ratio, and therefore have relative higher Alpha-2 concentrations.

## Discussion

The present study shows that NA innervation and Alpha adrenoceptor densities are markedly diverse among the thalamic nuclei bespeaking a considerable variety of NA mechanisms within the primate thalamus. We provide series of coronal maps displaying the distribution of NA axons and Alpha-adrenergic receptors in the macaque monkey thalamus, and offer quantitative data on Alpha-adrenergic receptor densities. The maps may serve as a reference tool for further neuroimaging, electrophysiological, and experimental studies focusing on NA in the primate thalamus.

The NA axonal innervation within the monkey thalamic nuclei is notably heterogeneous as depicted in Figures 3–5. Overall, midline nuclei and some intralaminar nuclei display the densest NA innervation. The medial MD nucleus also has significant NA innervation. Comparatively, the sensory relay nuclei are less innervated, with the lowest innervation present in the visual GLd nucleus. Ventral motor thalamic nuclei present a moderate to low NA innervation, with higher densities of NA axons in VAmc and VAvm (Figs 3, 4, and 6). These results are in general accordance with earlier fragmentary reports on NA innervation of the primate thalamus. Smith et al. (2006) and Vogt et al. (2008) described dense NET binding and DβH axons, respectively, in the medial thalamus and intralaminar nuclei; and Morrison and Foote (1986) showed a paucity of NA axons in the GLd nucleus and a denser innervation in the pulvinar-LP complex. A higher concentration of NA in VAmc, compared with the other ventral motor nuclei, was described by Oke et al. (1997) in the human thalamus. The present study surveyed all thalamic nuclei, adding significant further information to prior data.

Alpha-adrenergic receptors are, like NA axons, heterogeneously distributed throughout the thalamus, and, in general, matching the axonal density patterns. The receptor maps from this study concur with some limited early descriptions, like that of Palacios et al. (1987), in which the primate MD nucleus appears to have a relatively high density of Alpha-1 receptors when compared with other thalamic nuclei. The present work, however, provides considerably more detail, thanks to the comprehensive analysis of the distribution and the quantification of Alpha-1 and Alpha-2 receptors throughout the macaque monkey thalamic nuclei.

As described in Results, some nuclei display a nonmatching pattern of densities for NA axons and Alpha receptors. In CnMd, GM, Pul M, and Pul I, in particular, conspicuously high Alpha-adrenergic receptor densities occur in poorly or moderately NA innervated regions. In those nuclei, NA may preferentially modulate signal transmission through the Alpha rather than the Beta family of adrenergic receptors. The reverse pairing, a high NA axon innervation with low Alpha receptor densities (Pcn), may point out to a more prominent role for the Beta family receptors on noradrenergic signaling. Beta receptors have been previously shown in the human thalamus (Reznikoff et al. 1986) and, in rats, Beta-2 receptors have a high expression in intralaminar nuclei (Rainbow et al. 1984; Nicholas et al. 1993). Alternatively, or in addition, NA axons in Pcn could be coreleasing dopamine; this is plausible because dopamine axons are scarce in the Pcn, both in macaques and humans (Garcia-Cabezas et al. 2007), whereas Pcn has a high density of D2 receptors in humans (Rieck et al. 2004). Indeed, the release of dopamine by NA axons has been shown in the thalamus and in other brain regions (Beas et al. 2018; Ranjbar-Slamloo and Fazlali 2019); in fact, it may occur in thalamic nuclei other than the Pcn.

The other monoamines, dopamine (Garcia-Cabezas et al. 2007), adrenaline (Rico and Cavada 1998), and serotonin (Lavoie and Parent 1991), also have heterogeneous axon distribution patterns within the thalamus. The innervation patterns of those monoamines and NA show similarities and differences. The most outstanding similarity is the dense innervation in the midline nuclei by dopamine, NA, adrenaline, and serotonin. Likewise, in the posterior intralaminar nuclei, Pf is more densely innervated than CnMd by all monoamines. The anterior intralaminar Pcn nucleus is densely innervated by NA and serotonin but is devoid of dopamine and adrenaline innervations. The innervation within MD is quite heterogeneous for all monoamines, although specific patterns are present: MDm is highly innervated by all monoamines, whereas MDl and MDv additionally receive notable dopamine innervation. LP shows denser innervation by dopamine than by the other monoamines. GLd is very weekly innervated by the catecholamines but receives an appreciable serotonin innervation. Within the posterior group, Li and SG have high NA and serotonin innervations but low dopamine and adrenaline innervations. R is densely innervated by serotonin axons and heterogeneously innervated by dopamine and NA axons. Interestingly, dopamine and NA axons display similar patterns in R where the innervation is present in anterior and dorsal regions of the nucleus.

### Specificity of the Markers and Ligands Used and Methodological Discussion

DβH is the NA synthesizing enzyme from dopamine; therefore, it can also be present in adrenergic fibers. However, DβH immunoreactivity in adrenergic terminals is faint compared with that in NA terminals (Hökfelt et al. 1980), so that DβH has been widely used as a NA-axon marker in earlier studies (Ginsberg et al. 1993; Vogt et al. 2008). Besides, adrenaline fibers are scarce in the macaque monkey thalamus, except for the midline nuclei (Rico and Cavada 1998). The other NA axon marker used in the present study, NET, is a Na^+^ dependent transporter located presynaptically, and it is expressed only in noradrenergic cell bodies and axons (Lorang et al. 1994; Eymin et al. 1995; Schroeter et al. 2000). Additionally, we show here that both markers are specific, based on the anatomical selectivity of the immunostained neural bodies (Fig. 2), and in the consistent distributions of -ir axons using either marker (Fig. 3).

For Alpha adrenoceptor maps, we used in vitro autoradiography, which provides high-resolution images due to the beta-radiation of the [^3^H]-ligands. This enables the microscopic localization of the receptor subtypes sites in discrete areas, such as the layers of the cerebral cortex or the thalamic nuclei, including those of small size, that cannot be clearly dissected to perform binding studies in tissue homogenates (Manuel et al. 2015). The ligands used for Alpha-1 and Alpha-2 receptor autoradiography are specific: [^3^H]-prazosin binds mainly the Alpha-1 adrenoceptor (Zilles et al. 1993), and [^3^H]-UK-14,304 is a highly selective full Alpha-2 adrenoceptor agonist (Cambridge 1981; Loftus et al. 1984; Meana et al. 1989).

Autoradiography, however, does not allow distinguishing the cellular location of the mapped Alpha receptors: they might be located in presynaptic or postsynaptic neurons, or even in astrocytes (Stone and Ariano 1989; Fuxe et al. 2015). Nevertheless, autoradiography has great advantages, compared with immunohistochemistry, when receptors are analyzed: ligands only bind to the active receptors in the cell membranes, and therefore, autoradiography reveals the functional receptors, whereas immunohistochemistry would also show the internalized receptors, or the receptors present in a nonfunctional state due to posttranslational modifications. Additionally, receptor autoradiography is a quantitative technique, which allows gathering information about the concentration of the functional receptors in a particular region, and therefore, allows quantitative comparison between nuclei or subnuclei (Zilles et al. 1986; Zilles et al. 1988). Moreover, antibodies against adrenergic receptors are difficult to raise, and many commercial antibodies against Alpha receptors have proved to be unspecific (Jensen et al. 2009).

### Alpha-1/Alpha-2 Ratio in the Thalamic Nuclei

Alpha receptor ratios (see Table 3) are of interest to discuss NA function in the different thalamic nuclei. Alpha-1/Alpha-2 receptor ratio analysis reveals patterns with likely functional implications, yet unknown. Specifically, the anterior and ventral motor nuclei present a high ratio, and thus, relative high Alpha-1 receptors; the MD and the anterior intralaminar nuclei present a medium to high ratio; whereas the posterior intralaminar CnMd-Pf complex presents a low ratio, and thus, a relative high Alpha-2 receptor concentration. High Alpha-2 relative receptor concentrations also appear in the midline, pulvinar, and posterior nuclei; and in visual and auditory relay nuclei.

Alpha-1 and Alpha-2 receptors have different effects in the target cells (excitatory vs. inhibitory) and also different preferred localization (Alpha-1, mainly postsynaptic; Alpha-2, both presynaptic and postsynaptic) (Venkatesan et al. 1996; Nakadate et al. 2006). Therefore, differences in Alpha receptor ratios may be significant for neuronal activity in the presence of NA signaling, and therefore, are relevant to understand thalamic function in certain conditions, for example stress. Because Alpha-1/Alpha-2 relative ratio showed specific patterns in anterior and posterior thalamic nuclei (see final paragraph of Results), NA modulation may differ in anterior vs. posterior thalamus.

Agster et al. (2013) have argued that, in rat cortex, differences in varicosity densities (discussed below in this paper regarding the primate thalamus) allow for the possibility of differential effects on cognitive, sensory, and motor circuitries after the simultaneous release of NA as a result of locus coeruleus (LC) activation. We propose that not only differences in varicosity densities, but also receptor densities and Alpha receptor ratios, all together, frame a complex and precise mechanism that allows NA to modulate different systems, each in their own way, when this neurotransmitter is simultaneously released in several nuclei.

### Morphological Diversity of NA Axons

Assorted shapes in noradrenergic fibers have been previously described in the rat and macaque thalamus (Papadopoulos and Parnavelas 1990; Ginsberg et al. 1993). The varicosities have been studied using immunohistochemistry against the NA itself (Papadopoulos and Parnavelas 1990), against DβH (Ginsberg et al. 1993), and against NET (Schroeter et al. 2000).

We identified the NA varicosities using both markers; however, they were more evident when immunostained for DβH. Thus, anti-DβH immunohistochemistry was used here to describe the morphology and distribution of the varicosities in the various thalamic nuclei. Indeed, the enzyme DβH is located predominantly in the vesicles where NA is synthesized (De Potter et al. 1970; Pickel et al. 1996), and those vesicles are located preferentially in varicosities (Papadopoulos and Parnavelas 1990).

We observed the largest varicosities in the midline, intralaminar, and VAvm nuclei; NA varicosities in the remaining nuclei were overall smaller. Earlier studies in rat have shown a specific loss of thin or thick varicose axons after lesions of particular catecholaminergic nuclei (Lindvall et al. 1974; Ishikawa and Tanaka 1977). This suggests that the different morphologies of NA axons and varicosities in macaque thalamus might result from a different origin of the axons innervating each nucleus. It is known that the macaque thalamus receives projections from different noradrenergic groups, including LC, locus subcoeruleus, and A5 (Westlund et al. 1990).

Axons without varicosities are sparse in the macaque thalamus; they might be axons passing by to reach their target structures and not releasing NA as long as they are devoid of varicosities and terminals. We examined whether nonvaricose fibers were present in nuclei with high axonal NA innervation and low Alpha adrenoceptors concentrations, such as the Pcn. This was not the case: NA axons in Pcn, like in all thalamic nuclei, were predominantly varicose. Thus, the mismatch between high axonal innervation and low Alpha adrenoceptor densities is not explained by a high density of NA passing axons. This leads to hypothesize that NA released by the varicose axons in Pcn might be acting in this nucleus through Beta receptors.

### NA Modulation of Information in the Thalamus: Sensorimotor Gating and Cognition

Noradrenergic neurons increase their discharge during arousal, high vigilance, and attention, as well as in response to stimuli perceived as salient (Foote et al. 1980; Aston-Jones and Bloom 1981b). Studies on thalamic modulation by NA are limited, particularly in primates. It is relevant, however, that Coull et al. (2004) showed in humans that performance of a target detection task was decreased under dexmedetomidine treatment (an Alpha-2 adrenergic receptor agonist), and that this detrimental effect was reversed by exposure to white noise, involving higher order thalamic nuclei (pulvinar) selective activation.

Other studies have demonstrated a role for NA in the thalamus of rodents, in general related to attention and salience. NA sensory modulation in the thalamus has been demonstrated both in vitro and in vivo in the rodent visual (Rogawski and Aghajanian 1980; McCormick et al. 1991) and somesthetic systems (Castro-Alamancos and Calcagnotto 2001; Hirata et al. 2006; Devilbiss and Waterhouse 2011; Favero et al. 2012). This modulation mostly pertains to signal-to-noise ratios increase, receptive fields focus, facilitation of sensory-evoked responses, high-pass of corticothalamic signals, and/or potentiation of thalamo-cortical signals. In the macaque thalamus, primary visual transmission modulation by NA should be nominal, given the scarcity of NA axons and receptors present in GLd and in Pul L, connected to primary and secondary visual cortices. However, higher visual processing NA modulation might be taking place, given the outstanding Alpha receptor densities present in the Pul I nucleus, particularly in its posteromedial regions, which are connected to higher visual association cortical areas (Adams et al. 2000).

In the macaque somesthetic thalamus (VPM and VPL, in particular), moderate levels of NA axons and receptors are present, and therefore, a NA modulation may be present. However, some mechanisms may differ between species: for example, in rodents, NA-induced increase of signal-to-noise ratio in the VPM is due to the activation of R inhibition by NA (Hirata et al. 2006). Given that in the macaque ventral regions of R NA innervation is virtually absent and R shows low Alpha adrenoceptor concentrations (Figs 3, 4, and 6, Table 3), it is relevant to sort out the relative contributions of NA axons and their receptors within VPM and VPL themselves, since these may be more significant in primates than in rodents.

NA also plays a role in motor control (Grasso et al. 2006; Schiemann et al. 2015). In rats, NA depresses the firing rate of thalamic neurons both in VA and VL, acting mostly through Alpha-2 and less so through Beta receptors (Grasso et al. 2006). It remains to be demonstrated whether NA has comparable effects in the primate motor thalamic nuclei, where we show here an overall moderate density of NA axons and receptors; VAvm, VAmc, and area X, however, have high NA innervation and Alpha receptor densities (Figs 3, 4, and 6, Table 3).

Some evidence suggests the implication of thalamic NA in executive processing related to sensorimotor gating: NA agonists in the MD nucleus of rats disrupt prepulse inhibition (Alsene et al. 2011), a mechanism altered in some neuropsychiatric disorders. Analyzing in detail the role of NA in those mechanisms might be of particular interest in primates, considering the high density of NA axons and Alpha receptors in the MD nucleus, particularly in the medial sector of MD (Figs 3, 4, and 6, Table 3).

To understand NA mechanisms in the thalamus, it is necessary to dissect the relative contributions and relationships of NA release by axons and its action on adrenoceptors. Such relationships have been studied in the macaque monkey prefrontal cortex, where moderate levels of NA signaling couple with optimal performance in tests of working memory, whereas too low or excessive NA impairs the performance. This occurs because of a differential activation of NA receptor subtypes within the prefrontal cortex, which follows an inverted U-shaped function: high-affinity Alpha-2 receptors in the prefrontal cortex are activated under moderate levels of NA, and this improves working memory performance, whereas Alpha-1 receptors are activated in the prefrontal cortex under high concentrations of NA, and its activation by means of agonists elicits a stress-like impairment in working memory (Berridge and Spencer 2016). It would be valuable to test if a comparable inverted U-shape response to NA occurs throughout the thalamic nuclei; as well as if this is related to the Alpha-1/Alpha-2 receptor ratio. An interesting nucleus to test this hypothesis is CnMd, where there is a very high Alpha-2 receptor concentration, compared with the other thalamic nuclei (Table 3).

### Thalamic NA and Cortico-Thalamo-Striatal Circuits

The present data show that the nuclei with the highest densities of NA axons and Alpha receptors are those related to thalamo-striatal circuits: midline nuclei, intralaminar Pcn nucleus, and CnMd-Pf complex (Figs 3, 4, and 6, Table 3). Within the CnMd-Pf complex, axons and receptors density differences are notable: the highest innervation and receptor densities are in Pf, particularly its caudal region; whereas CnMd displays an average innervation. The thalamo-striatal projections are strongly segregated topographically (Parent et al. 1983; Gimenez-Amaya et al. 1995; Galvan et al. 2016). The highly NA-innervated thalamic regions (midline, Pcn, Pf) are those projecting to the limbic and associative striatum, and not so much to the sensorimotor striatum.

Within the ventral motor group, VAvm and VAmc are highly innervated by NA; these nuclei are part of limbic and associative cortico-thalamo-striatal circuits (Ilinsky et al. 1985; Cavada et al. 1995; McFarland and Haber 2001, 2002). Likewise, Li, which also receives a high NA-innervation, is connected to the associative striatum (Parent et al. 1983).

It is worth mentioning that, unlike almost all the central nervous system regions, which receive NA innervation to a greater or lesser extent, the striatum and globus pallidus are virtually devoid of NA axons, the exception being some regions in the nucleus accumbens (Berridge et al. 1997; Smith et al. 2006). The particularly dense NA innervation within the thalamic nuclei projecting to the striatum may perform an indirect noradrenergic modulation of basal ganglia activity, particularly in the circuits related to limbic and associative processing.

### Thalamic NA in the Mediodorsal Nucleus

MD is a higher order thalamic nucleus that receives driver inputs from layer V of the prefrontal cortex (Giguère and Goldman-Rakic 1988; Rovo et al. 2012; Sherman and Guillery 2013), and in turn projects mostly to the prefrontal cortex (Barbas et al. 1991). MD is composed of several subdivisions, differing on myeloarchitecture, cytoarchitecture, and chemoarchitecture (Olszewski 1952; Cavada et al. 1995), and connected with different prefrontal regions (Russchen et al. 1987; Ray and Price 1993; Cavada et al. 1995; Cavada et al. 2000; Erickson and Lewis 2004; Xiao et al. 2009). The most NA innervated sector is MDm which also has high concentrations of adrenoceptors, among the highest within the thalamus (Figs 3–5, Table 3). MDm is connected to the orbitofrontal cortex (Russchen et al. 1987; Ray and Price 1993; Cavada et al. 1995; Cavada et al. 2000) and the amygdala (Porrino et al. 1981; Russchen et al. 1987; Timbie et al. 2020), and consequently, it is the most “limbic” sector within MD. MD also receives input from basal ganglia output nuclei (substantia nigra pars reticulata and ventral globus pallidus) (Russchen et al. 1987; Erickson et al. 2004); MDm, in particular, receives input from the ventral globus pallidus, the output nucleus of the basal ganglia limbic circuit. In fact, MDm is necessary in macaques to perform cognitive tasks that require learning and/or adaptive decision-making (Browning et al. 2015; Chakraborty et al. 2016). Altogether, the prominent NA innervation and adrenoceptors in MD suggest a role for NA in the limbic and associative circuits involving the thalamus, the prefrontal cortex, particularly the orbitofrontal cortex, and the basal ganglia.

MD, unlike other thalamic nuclei that establish contacts only with particular subregions of R, sends branching axons that extend into wide regions of R (Zikopoulos and Barbas 2007). This suggests that MD activity may influence other thalamic nuclei through its widespread interactions with R; in other words, NA modulation of MD might have extensive effects on the activity of other thalamic nuclei.

### Thalamic NA and Consciousness States

Sleep may be considered a physiological diminished state of consciousness (Pal and Mashour 2011), which broadly consists of 2 different phases {non-REM {NREM} and REM sleep} characterized by electroencephalography patterns, behavioral states, and neurochemical cyclic variations including noradrenergic activity: LC neurons discharge fastest during waking, slow down during NREM sleep, and reach their lowest activity rates during REM sleep (Aston-Jones and Bloom 1981a). During NREM sleep, theta waves appear in the electroencephalogram; these waves are in part generated by thalamic relay neurons due to their intrinsic electrophysiological properties. Serotonin and NA regulate this process (McCormick and Pape 1990). Also, NREM sleep spindles are a consequence of the interaction between R nucleus intrinsic activity and thalamic relay cells (Steriade et al. 1985) and are modulated by NA levels in R (McCormick and Wang 1991; Destexhe et al. 1994). We found only a moderate-to-low NA innervation in the macaque R, which is even almost absent in posterior and ventral regions (Figs 3, 4, and 5*E*). This is in contrast with the high NA innervation in R previously described in monkeys (Morrison and Foote 1986). The present account of NA innervation in R is based on data from 2 markers of the NA phenotype, DβH and NET, which gave similar results (Fig. 3), and on a complete survey of the R nucleus. We show that anterior and dorsal R regions are the most innervated by NA (Figs 3, 4, and 5*E*). We put forward that those NA-innervated regions of R, which are at the crossroads of fronto-thalamo-frontal circuits (Zikopoulos and Barbas 2006, 2007), are specifically involved in sleep spindles mechanisms. Alpha receptors in R were almost absent (Fig. 6, Table 3). Beta receptors, however, are abundant in the R nucleus of the rat (Nicholas et al. 1993) but not in the human (Reznikoff et al. 1986). It remains to be explored whether Beta adrenoceptors are present in the primate R nucleus and may account for a role of NA in NREM sleep spindles.

The present study shows that the midline and intralaminar thalamic nuclei receive a strong innervation by NA axons. Recent studies have demonstrated in rats that midline nuclei present slow waves earlier than the cortex and the thalamic sensory relay nuclei (Baker et al. 2014; Gent et al. 2018a). In human patients, deactivation at sleep onset occurs earlier in the higher order pulvinar nucleus than in the cortex (Magnin et al. 2010). This evidence led some authors to hypothesize that midline and higher order nuclei drive the global cortical state during sleep, whereas slow waves in primary sensory thalamus appear later (Gent et al. 2018b). It is possible that NA dynamics in midline, intralaminar, and possibly higher order thalamic nuclei play a critical role during sleep onset and early sleep stages.

Anesthesia is a diminished state of consciousness, pharmacologically induced, and reversible. It is a state clearly distinguishable from sleep; however, some authors suggest that the effects of anesthesia may be mediated through the same subcortical brain network that controls sleep–wake states (Franks 2008; Pal and Mashour 2011). This network includes the thalamus and the locus coeruleus.

The thalamus and thalamo-cortical circuits are crucial for anesthetic-induced unconsciousness (Alkire and Miller 2005). Dexmedetomidine, an Alpha-2 adrenergic receptor agonist, is among the anesthetic compounds currently used. Recently, it has been demonstrated that dexmedetomidine-induced unconsciousness is mediated by thalamo-cortical disconnection, whereas cortico-cortical connections remain functional (Akeju et al. 2014). It awaits to be examined whether the disruption of thalamo-cortical connections is just due to the binding of dexmedetomidine to presynaptic Alpha-2 receptors in the LC neurons, including their axons, or if binding of dexmedetomidine to postsynaptic Alpha-2 receptors in the thalamus has a particular role in the mechanisms of unconsciousness. Of particular interest would be to elucidate the role of the pulvinar nucleus, given that pulvinar deactivation precedes cortical deactivation in sleep onset in humans (Magnin et al*.* 2010), and that this nucleus shows one of the highest Alpha-2 receptor concentrations in the thalamus (Figure 6*G*″, Table 3).

Coma, vegetative state, and minimally conscious state after brain injury include decreased consciousness. Damage to the intralaminar thalamic nuclei, which receive a dense NA innervation (Figs 3–6, Table 3), is reckoned as one of the causes for the pathological loss of consciousness (Castaigne et al*.* 1981; Lin et al*.* 2017). The restoration of functional connectivity between the intralaminar thalamic nuclei and prefrontal and anterior cingulate cortices has been linked to the recovery of consciousness (Laureys et al*.* 2000). Also, intralaminar thalamic deep brain stimulation has been shown to be beneficial to treat brain injury-induced unconsciousness; such stimulation aimed to elicit excitation of intralaminar projections to the cortex and striatum (Giacino et al*.* 2012). Altogether, a role for intralaminar nuclei in consciousness seems evident, and their dense NA innervation may play key roles in consciousness maintenance.

### Thalamic NA in Neurological and Psychiatric Disorders

NA levels in the thalamus of PD patients are markedly decreased (Pifl et al*.* 2012). The NA loss is generalized but is greatest in motor, limbic, association, and intralaminar nuclei and less so in the sensory relay nuclei. Dysregulation of thalamic NA in PD may contribute to motor and nonmotor symptoms. It is relevant that executive dysfunction in PD improves after treatment with the NA reuptake inhibitor atomoxetine (Marsh et al*.* 2009; Rae et al*.* 2016).

Numerous evidence couple the thalamus with various neuropsychiatric disorders, such as autism (Iidaka et al*.* 2019), schizophrenia (Clinton and Meador-Woodruff 2004), PTSD (Liberzon and Abelson 2016), and depression (Zhang et al*.* 2018). Also, some studies suggest a role for NA in some of those conditions (Hendrickson and Raskind 2016; Maletic et al*.* 2017). Most psychiatric disorders happen with dysregulation of limbic, cognitive, arousal, and valence systems; often associated with prefrontal dysfunction. Since most of the highly NA-innervated nuclei in the thalamus are related to limbic circuits or to circuits involving the prefrontal cortex (e.g., MD nucleus), disease mechanisms and treatments might involve the thalamus, and particularly the thalamic NA system. For example, PTSD is associated with impaired prefrontal regulation of emotion (Liberzon and Abelson 2016; Fitzgerald et al*.* 2018), and prazosin, an Alpha-1 adrenergic receptor antagonist, is commonly used to treat the condition (Boehnlein and Kinzie 2007; Simon and Rousseau 2017). We have shown high Alpha-1 densities in the midline nuclei, with important limbic connections, and in the MD nucleus, connected with the prefrontal cortex (Fig. 6, Table 3). It is conceivable that binding of prazosin to Alpha-1 thalamic adrenoceptors in those nuclei may play a role in the therapeutic effect of prazosin.

As mentioned before, NA in the thalamus (particularly in MD) is involved in prepulse inhibition (Alsene et al*.* 2011). Deficient prepulse inhibition is used as a model of altered sensorimotor gating in conditions like schizophrenia and Tourette’s syndrome. Clonidine, an Alpha-2 adrenoceptor agonist, has been shown to normalize sensorimotor gating in patients with schizophrenia (Oranje and Glenthoj 2013), and it is commonly used to treat Tourette’s syndrome, especially in comorbidity with ADHD. Given the evidence of thalamic involvement in prepulse inhibition (Alsene et al*.* 2011), an effect of clonidine through the thalamus is feasible.

### Concluding Remarks

NA is a modulatory neurotransmitter with important functions in the brain. In the present work, we have shown the distribution of NA axons and Alpha adrenoceptors throughout the macaque monkey thalamic nuclei. We have demonstrated a widespread distribution of NA axons and receptors throughout the thalamus; with the highest NA axon and receptor densities in nuclei related to limbic, arousal, and cognitive mechanisms; as well as in nuclei involved in thalamo-striatal circuits. These findings lead us to propose a key role for thalamic NA in modulating limbic, attentional, cognitive, and executive functions. The alteration of thalamic NA might disrupt these functions. Treatments of brain conditions with Alpha adrenoceptors agonists or antagonists should take into account the thalamic NA system for a complete understanding of their mechanisms of action. The present work may serve as a reference tool for future neuroimaging, electrophysiological and further studies focusing on NA in the primate thalamus.

## Funding

Chair in Neuroscience UAM-Fundación Tatiana Pérez de Guzmán el Bueno (C.C and I.P.-S.); the German Federal Ministry of Education and Research (01GQ1902 to N.P.-G.); the European Union’s Horizon 2020 Research and Innovation Programme (785907 (Human Bain Project SGA2) and 945539 (Human Brain Project SGA3) to N.P.-G. and K.Z.); Boehringer Ingelheim Fonds (I.P.-S.); Universidad Autónoma de Madrid (I.P.-S.).

## LIST OF ABBREVIATIONS

**AChE**: acetylcholinesterase
**AD**: anterodorsal nucleus
**ADHD**: attention deficit hyperactivity disorder
**AM**: anteromedial nucleus
**AMdc**: anteromedial nucleus densocellular part
**AP**: anteroposterior distance to the interaural coronal plane
**AV**: anteroventral nucleus
***Bc***: brachium conjunctivum
***Bcs***: brachium of superior colliculus
**B**_**max**_: receptor concentration in an area of interest (in fmol/mg of protein)
**B**_**maxSUCC**_: succinimidyl calculated concentration in an area of interest
**B**_**maxWM**_: succinimidyl calculated concentration in *corona radiata*
**BSA**: bovine serum albumin
**Cdc**: central nucleus–densocellular part
**CeM**: central medial nucleus
**Cif**: central nucleus–inferior part
**Cim**: central nucleus–intermediate part
**CL**: central lateral nucleus
**Clc**: central nucleus—latocellular part
**C**_**max**_: maximum concentration of radioactivity in an autoradiogram
**CnMd**: centromedian nucleus
**Cs**: central superior nucleus
**Csl**: central nucleus—superior lateral part
***Ct***: corticotectal tract
**DAB**: diaminobenzidine
**DβH**: dopamine beta hydroxylase
**fmol/mgP**: femtomole *per* mg of protein
**GLd**: dorsal lateral geniculate nucleus
**GLv**: ventral lateral geniculate nucleus
**GM**: medial geniculate nucleus
**GMmc**: medial geniculate nucleus—magnocellular part
**GMpc**: medial geniculate nucleus—parvocellular part
**III**: third ventricle
**-ir**: immunoreactive
**IV**: fourth ventricle
**K**_**D**_: equilibrium dissociation constant
**L**: incubation concentration of ligand
**LC**: Locus coeruleus
**LD**: lateral dorsal nucleus
**Li**: nucleus limitans
***Lme***: external medullary lamina
**LP**: lateral posterior nucleus
**MD**: mediodorsal nucleus
**MDl**: mediodorsal nucleus—lateral sector
**MDm**: mediodorsal nucleus—medial sector
**MDmf**: mediodorsal nucleus—multiformis sector
**MDv**: mediodorsal nucleus—ventral sector
**MDp**: mediodorsal nucleus—posterior sector
**NA**: noradrenaline
**NET**: noradrenaline transporter
**PAG**: periaqueductal gray matter
**PB**: phosphate buffer
***Pcm***: middle cerebellar peduncle
**Pcn**: paracentral nucleus
**PD**: Parkinson’s disease
**PET**: positron emission tomography
**Pf**: parafascicular nucleus
**Po**: posterior nucleus
**Pt**: paratenial nucleus
**PTSD**: posttraumatic stress disorder
**Pul I**: inferior pulvinar nucleus
**Pul L**: lateral pulvinar nucleus
**Pul M**: medial pulvinar nucleus
**Pul O**: oral pulvinar nucleus
**Pv**: paraventricular nucleus
**Pvc**: paraventricular nucleus—caudal part
**R**: reticular nucleus
**Re**: nucleus reuniens
**S**_**A**_: specific activity of the ligand
**SC**: superior colliculus
**SCo**: nucleus subcoeruleus
**Sg**: suprageniculate nucleus
***Sm***: stria medullaris
**TBS**: tris buffer saline
***Thi***: habenulo-interpeduncular tract
***Tmt***: mamillothalamic tract
***Tp***: pyramidal tract
**UK-14304**: 5-Bromo-N-(4,5-dihydro-1H-imidazol-2-yl)-6-quinoxalinamine
**VA**: ventral anterior nucleus
**VAmc**: ventral anterior nucleus—magnocellular part
**VAmcd**: ventral anterior nucleus –magnocellular dorsal part
**VApc**: ventral anterior nucleus—parvocellular part
**VAvm**: ventral anterior nucleus—ventromedial part
**VLc**: ventral lateral nucleus—caudal part
**VLm**: ventral lateral nucleus—medial part
**VLo**: ventral lateral nucleus—oral part
**VLps**: ventral lateral nucleus–pars postrema
**VPI**: ventral posterior inferior nucleus
**VPLc**: ventral posterior lateral nucleus–caudal part
**VPLo**: ventral posterior lateral nucleus–oral part
**VPM**: ventral posterior medial nucleus
**VPMpc**: ventral posterior medial nucleus–parvocellular part
**X**: area X
**ZI**: zona incerta
